# Recent advances in understanding antiphospholipid syndrome

**DOI:** 10.12688/f1000research.9717.1

**Published:** 2016-12-22

**Authors:** Maria Laura Bertolaccini, Giovanni Sanna

**Affiliations:** 1 Academic Department of Vascular Surgery, Cardiovascular Division, King’s College London, London, UK; 2 Louise Coote Lupus Unit, Guy's and St Thomas' NHS Foundation Trust, London, UK

**Keywords:** antiphospholipid syndrome, APS, Hughes Syndrome

## Abstract

Antiphospholipid syndrome (APS), also known as Hughes Syndrome, is a systemic autoimmune disease characterized by thrombosis and/or pregnancy morbidity in the presence of persistently positive antiphospholipid antibodies. A patient with APS must meet at least one of two clinical criteria (vascular thrombosis or complications of pregnancy) and at least one of two laboratory criteria including the persistent presence of lupus anticoagulant (LA), anticardiolipin antibodies (aCL), and/or anti-b2 glycoprotein I (anti-b2GPI) antibodies of IgG or IgM isotype at medium to high titres in patient’s plasma. However, several other autoantibodies targeting other coagulation cascade proteins (i.e. prothrombin) or their complex with phospholipids (i.e. phosphatidylserine/prothrombin complex), or to some domains of β2GPI, have been proposed to be also relevant to APS. In fact, the value of testing for new aPL specificities in the identification of APS in thrombosis and/or pregnancy morbidity patients is currently being investigated.

## Introduction

Antiphospholipid syndrome (APS), also known as Hughes Syndrome, is a systemic autoimmune disease characterized by thrombosis and/or pregnancy morbidity in the presence of persistently positive antiphospholipid antibodies ^1^. When APS was first described, it was in the presence of systemic lupus erythematosus (SLE) ^2^; however APS is now accepted to be a primary autoimmune syndrome with other accompanying characteristics, such as thrombocytopenia, seizure disorder, cognitive dysfunction, livedo reticularis, and renal vasculopathy, being frequent in the absence of the main clinical manifestations of thrombosis and pregnancy complications ^3^.

In 1999 definitive classification criteria for APS were published in an international consensus statement ^4^ and subsequently revised in 2006 ^1^. A patient with APS must meet at least one of two clinical criteria (vascular thrombosis or complications of pregnancy) and at least one of two laboratory criteria including the persistent presence of lupus anticoagulant (LA), anticardiolipin antibodies (aCL) and/or anti-β2 glycoprotein I (anti-β2GPI) antibodies of IgG or IgM isotype at medium to high titres in patient’s plasma.

While it is widely accepted that the LA is the most important predictor for thrombosis ^5– 7^, several other autoantibodies targeting other coagulation cascade proteins (i.e. prothrombin) or their complex with phospholipid (i.e. phosphatidylserine/prothrombin complex), or to some domains of β2GPI, have been proposed to be relevant to APS ^8^. In fact, the value of testing for new aPL specificities in the identification of APS in thrombosis and/or pregnancy morbidity patients is currently being evaluated, which will be especially useful for those with recurrent negative results in present tests ^9^.

## New aPL specificities

### Antibodies directed to the domain I of the β2GPI

β2GPI was identified as a primary target of autoantibodies in patients with APS ^10^. β2GPI is a single-chain protein containing five repeating sequences or domains. Domain V is essential for binding to anionic phospholipid membranes, whereas domain I sticks out into the extracellular space where interactions with other proteins/antibodies can take place ^11^. The development of recombinant domain specific β2GPI molecules by Iverson *et al*. in 1998 ^12^ steered us towards a better understanding of the specific role of the autoantibodies to each of the five β2GPI domains. Several studies have detected antibodies recognizing various domains of β2GPI ^13^. However, anti-domain I (anti-DI) antibodies were frequently found to be highly associated with clinical symptoms and therefore focused upon ^14, 15^.

In their 2005 study, de Laat *et al*. reported that patients testing positive for anti-DI had a higher thrombosis risk ^14^. Antibodies recognizing epitope G40-R43 on the domain I of β2GPI caused LA and strongly correlated with thrombosis ^14^. A larger, multicentre study in 2009 looked at a large cohort of anti-β2GPI positive patients, showing that those patients who were IgG anti-DI positive had a 3.5 fold increase in the risk of developing vascular thrombosis and a 2.4 fold increase in the risk of developing pregnancy morbidity when compared to those who tested negative for IgG anti-DI ^16^. Using inhibition assays, Banzato *et al*. demonstrated that high-risk patients, those bearing triple aPL positivity for aCL, LA and anti-β2GPI, are those with substantially greater titre of circulating anti-DI antibodies. Those with double and single positivity showed low titre or absence of anti-DI antibodies ^17^. Conversely, when tested on 326 patients with SLE, of whom 164 had a history of thrombosis, Akhter *et al.* failed to find an association between anti-DI and these events ^18^.

The domain profile of anti-β2GPI antibodies has also been explored in a large cohort of patients. While neither anti-DI nor anti-DIV/V antibodies were found to be associated with thrombotic events or obstetric morbidity, Andreoli *et al.* suggested that utilizing the ratio of anti-DI/anti-DIV/V could be useful as a biomarker for APS, identifying “pathogenic” from “non-pathogenic” anti-β2GPI ^15^. A recent study in aCL and/or aβ2GPI positive patients suggests that the added finding of anti-DI positivity makes it three to five times more likely to confirm APS. Positivity for IgG or IgA (but not IgM) anti-DI increased the strength of association between aCL/aβ2GPI and thrombotic manifestations in APS ^19^.

Anti-DI antibodies have also been reported in pediatric populations. Wahezi *et al.* reported a prevalence of IgG anti-DI of 25.1% in children with SLE. However, only seven children had thrombosis, failing to ascertain a positive correlation ^20^. In a study on 64 APS patients and 57 children born to mothers with systemic autoimmune diseases, Andreoli *et al. s*howed a high prevalence of anti-DI in APS while there was a low anti-DI frequency reported in anti-β2GPI positive healthy children ^21^.

A direct demonstration of the pathogenic effect of anti-DI antibodies has been recently shown using a human monoclonal IgG (MBB2), the infusion of which brought about fetal losses in pregnant mice and blood clots in rat mesenteric microcirculation following priming with lipopolysaccharide (LPS) ^22^. Interestingly, a variant of this antibody, lacking the CH2 domain (MBB2DΔCH2), was effective in preventing blood clot formation and fetal loss induced by aPL ^22^. A recombinant human domain I has also been shown to inhibit the ability of polyclonal human IgG from a patient with APS to cause thrombosis or to enhance tissue factor activity in an animal model ^23^. Using polyclonal IgG from patients with APS, anti-domain I-rich IgG significantly enhanced prothrombotic ability *in vivo* compared with anti-domain I-poor or NHS-IgG, suggesting that the ability of human APS-derived IgG to cause thrombosis in mice is concentrated in the anti-domain I-rich fraction ^24^.

A novel approach for developing therapy for APS has shown that tolerogenic dendritic cells specific for domain-I of the β2GPI molecule may have potential in attenuating experimental APS in a murine model, via acceleration of the differentiation of CD4 ^+^ T cells to Treg cells, decreased proinflammatory cytokine production, and increased anti-inflammatory cytokine expression (IL-10 and TGFβ) ^25^.

### Antibodies to prothrombin

Prothrombin (factor II) is an important antigenic target for aPL in APS. Prothrombin is a vitamin K-dependent single-chain glycoprotein of 579 amino acid residues with a molecular weight of 72-kDa. It circulates in normal plasma at a concentration of approximately 100 μg/ml ^26^. Antibodies directed to human prothrombin (aPT) and the complex of phosphatidylserine/prothrombin (aPS/PT) are detected by ELISA and have been strongly associated with APS ^27^. While the presence of these antibodies have been shown to correlate in some cases ^28^, it seems that aPT and aPS/PT belong to different populations of autoantibodies ^9^.

A systematic review of the literature including 6000 patients and 1400 controls has been recently reported ^27^. aPS/PT was shown to represent a stronger risk factor for thrombosis, both arterial and/or venous, than aPT, with an odds ratio (OR) of 5 ^27^. Data from our group and others suggest that the risk of thrombosis progressively increases with the increase in number of positive aPL tests ^29– 32^. Recently, we showed that testing positive for all three antibodies—LA, anti-β2GPI and aPS/PT— was the best diagnostic indication of APS ^33^. In addition, when compared with double or single positivity, this triple combination showed a stronger correlation with clinical events (thrombosis and/or pregnancy loss).

The mechanisms underlying the procoagulant properties of antibodies to prothrombin are not known; currently two are being postulated: a) indirect; through humoral regulators of coagulation (i.e. prothrombin) or b) direct; engaging/activating cell receptors. An isolated report suggests that polyclonal antibodies from patients with antiprothrombin antibodies might act on a ‘target’ molecule expressed at the endothelial cell surface ^34^, although this is as yet uncharacterised. Tissue factor production induced by aPS/PT in procoagulant cells is reported to occur predominantly via activation of the p38 mitogen-activated protein kinase (MAPK) pathway ^35^, similar to the mechanisms implicated in anti-β2GPI-induced cell activation ^36^. In the mouse, active immunisation with prothrombin is associated with increased thrombosis, supporting a role for antibodies to prothrombin in thrombus formation ^37^. In addition, mice treated with IS6 (a mouse monoclonal antiprothrombin antibody) show thrombi that are larger and persist longer than in mice injected with control antibody ^34^.

## Pathogenic mechanisms of aPL

Despite our incomplete understanding of APS pathogenesis, the major facets have been defined in recent years. Thrombosis, a key feature of the disease, can be the result of various mechanisms, including endothelial cells, monocytes, platelets, coagulation, and complement pathways, as well as blocking of the fibrinolytic and anticoagulation pathways. The conventional understanding is that aPL antibodies bind to receptors on target cells, causing their activation and leading to thrombosis in large vessels ^38^. A number of processes have been implicated as effectors of a prothrombotic state in APS. These include: the generation of tissue factor ^39, 40^; complement activation ^41– 43^; activated platelet-enhanced endothelial activation ^44, 45^; monocyte protease receptor activation ^46^; and the generation of DNA nets by neutrophils ^44, 45^.

aPL have been proposed to bind to cellular membranes via various different receptors, including annexin A2 ^47– 50^, apolipoprotein E receptor 2 (ApoER2) ^51– 53^, low-density-lipoprotein receptor (LDL-R) ^54^, megalin ^55^, Toll-like receptors 2 ^56, 57^ and 4 ^50, 58^, and the very-LDL-R and P-selectin glycoprotein (GP) ligand-1. It has also been shown that β2GPI is able to directly bind to the platelet adhesive receptor GPIbα ^59, 60^ and the platelet factor4 (PF4) ^61^.

Antibody binding to aPL receptors on target cells activates intracellular mediators like nuclear factor kappa B (NF-κB) and p38MAPK ^62^.

aPL have also been shown to activate the phosphatidylinositol 3-kinase (PI3K)–AKT pathway. Activation of this signaling cascade engages the mammalian target of rapamycin (mTOR), a kinase modulating cellular growth, proliferation and survival ^63^. Polyclonal aPL from APS patients induced a marked increase in S6RP and AKT (Ser473) phosphorylation, two of the components of the mTOR pathway, mediating intimal hyperplasia and chronic vasculopathy often seen in APS ^63^.

## aPL and the Coagulation System

aPL have been reported to inhibit the anticoagulant properties of activated protein C (APC) ^64, 65^, impair fibrinolysis ^66– 69^, reduce tissue factor pathway inhibitor (TFPI) activity ^70, 71^ and β2GPI-thrombin interaction ^72, 73^, and disrupt the annexin A5 anticoagulant shield ^74– 77^. The binding of aPL to β2GPI diminishes β2GPI complement regulatory function with the consequent impaired clearance of apoptotic cells ^78^.

## aPL as risk factors for thrombosis: Scoring Systems in APS

One of the unexplained matters in APS is why some patients develop thrombotic events while others present with morbidity in pregnancy. While a minority of patients may also develop a life-threatening “catastrophic” form of APS with multiple organ involvement and a high death rate, others never develop any aPL-related manifestation.

In this context, assessing the patient risk of developing an aPL-related manifestation is crucially important for physicians. Three score systems have been formulated to quantify the risk of thrombosis/obstetric events in APS ^31, 79, 80^.

In 2011, a risk model for APS diagnosis was developed based on patient positivity for aPL along with their titre and the results obtained for LA investigation ^31^. Probability estimates for diagnosis of APS were obtained using logistic regression equations and the authors demonstrated that multiple aPL positivity, primarily the triple association of LA, aCL and anti-β2GPI, increased the risk of APS. LA was shown to be the strongest aPL associated with the diagnosis of APS.

In an attempt to quantify the risk based on the aPL profile, Otomo *et al.* ^79^ designed the “antiphospholipid score” or aPL-S. The aPL profiles were analyzed using six ELISAs (IgG/IgM aCL, IgG/IgM anti-β2GPI, and IgG/IgM aPS/PT) and five clotting assays for LA. An algorithm generated this score, with each assay being assigned different points weighted on the relative risk of having a clinical manifestation of APS. The prevalence of APS manifestations increased with the increasing aPL-S, suggesting that the aPL-S could serve as a marker of the “probability” of APS and a valuable tool for predicting thrombosis. An independent validation in a separate cohort of 211 consecutive SLE patients confirmed the aPL-S correlation with a history of thrombosis or pregnancy loss ^81^.

Our newly developed alternative score for APS diagnosis (Global APS score or GAPSS) is based on independent thrombosis and pregnancy loss risk factors ^80^. This score accounts for established cardiovascular risk factors and the autoimmune antibodies profile in addition to the aPL profile (criteria aPL ^1^ and non-criteria aPL ^82^). We developed and validated the score system in a SLE cohort. The analysis included data on clinical manifestations, conventional cardiovascular risk factors, aPL and autoimmune profile (including ANA, ENA and anti-dsDNA, among others). Weighted points proportional to the β-regression-coefficient values were assigned to each independent risk factor identified by multivariate analysis. Validation was performed in a second cohort of patients showing statistically significant higher values of GAPSS in those with a clinical history of thrombosis and/or pregnancy loss when compared to those without events.

When applied in a prospectively followed-up cohort of SLE patients, an increase in the GAPSS during this follow up was found to be associated with a 12-fold increase in the risk of vascular events. In detail, an increase of more than 3 GAPSS points seemed to have the best risk accuracy for vascular events with a hazard ratio of 48 ^83^. 

This score was also applied to a cohort of primary APS, higher values of GAPSS were seen in APS patients who experienced thrombosis when compared to those with previous pregnancy loss alone. In addition, GAPSS was able to discriminate patients who experienced recurrent thrombotic events from those without recurrences ^84^.

This score was independently validated by two groups. Zuily *et al.* ^85^ evaluated the validity of the GAPPS to predict thrombosis in a prospective multicentre cohort study. GAPSS values were significantly higher in patients who experienced a thrombotic event when compared to those without with a reported GAPSS above 16 as a significant predictor of thrombosis in this population. Oku *et al*. ^86^ confirmed that GAPSS can be successfully used to quantify risk in an independent cohort of patients with autoimmune diseases. GAPSS correlated with a history of APS symptoms, particularly with thrombosis, implying it can be used as an appropriate quantitative marker for APS.

## Classification vs. diagnostic criteria

As stated above, in 1999, definitive classification criteria for APS were published in an international consensus statement ^4^ and a subsequent revision was made in 2006 ^1^. A patient with APS must meet at least one of two clinical criteria (vascular thrombosis or complications of pregnancy) and at least one of two laboratory criteria including the persistent presence of lupus anticoagulant (LA), anticardiolipin antibodies (aCL) and/or anti-β2GPI antibodies of IgG or IgM isotype at medium to high titres in patient’s plasma.

These classification criteria are aimed at identifying well defined, relatively homogeneous group of patients, all sharing key features of the condition, as they do not reflect the different features of the disease, as diagnostic criteria should ^87^. To date, there are no diagnostic criteria available for APS and, therefore, even with a lack of ‘essential’ or ‘key’ features, clinicians should be encouraged to consider the diagnosis in the presence of ‘minor’ features, providing other causes have been ruled out.

## aPL carriers

Overall data from available studies suggest that asymptomatic aPL carriers bear a 0–2.8% annual risk of developing a thrombotic event ^88^. While the presence of aPL is necessary but not sufficient to provoke a thrombotic event, the “second hit” hypothesis suggests that an additional trigger is needed to initiate a vascular event in aPL carriers.

An early study from 1998 ^89^ evaluated the prevalence of thrombosis in aCL positive patients with SLE. The authors reported that 52% of aCL carriers developed a thrombotic event during the 10-year follow up, opening the question on the importance of these antibodies as risk factors for thrombosis. From then, few other studies have estimated the incidence of thrombosis in asymptomatic carriers with aPL. A total of 178 asymptomatic aPL carriers without underlying autoimmune diseases underwent a 3-year prospective observational cohort study and no thrombotic events were reported during follow up ^90^. The APLASA study, a randomized, double-blind, placebo-controlled trial investigating the efficacy of low-dose aspirin (LDA) as primary prevention of thrombotic events showed a low incidence of thrombosis in aPL carriers, events occurring in all but one of the cases, in the presence of concomitant thrombosis risk factors and/or systemic autoimmune disease at the time of thrombosis ^91^. A prospective study identified hypertension and LA as independent risk factors for a first thrombotic event in asymptomatic aPL carriers ^92^.

A recent study evaluating the efficacy and safety of LDA vs. LDA plus low-intensity warfarin in the primary thrombosis prevention of aPL-positive patients with SLE and/or obstetric morbidity reported an incidence of 1.8 events/100 person-years in the randomized group ^93^. Interestingly, this incidence was increased to 4.9 events/100 person-years in the observational arm with hypertension being the most frequent additional risk factor.

Evidence shows that patients with more than one positive test, and particularly those with all three positive aPL tests (referred to as triple positive), are those with a strong association with clinical events ^29, 30^. Therefore, aPL carriers should be risk-stratified according to the aPL status, the presence of other cardiovascular risk factors that should be closely monitored and controlled whenever possible, and the concomitance of other systemic autoimmune diseases.

## Conclusions

Studies are underway to establish the value of testing for new aPL specificities in the identification of APS in patients with thrombosis and/or pregnancy morbidity, particularly in those for whom repeated testing produces negative results with currently available methods. While their clinical importance and mechanisms of action are far from being fully explored, available data suggest that the presence of these other aPL, particularly anti-DI and aPS/PT antibodies, are useful for risk stratification.

Ongoing research focuses on cell receptors and intracellular signaling pathways involved in the cell activation mediated by aPL. The clarification of these mechanisms is crucial to a better understanding of pathogenesis of APS. Although some controversial data still exist in regards to new specificities, most of the available reports support the association between aPS/PT, and to a lesser extent anti-DI, and the clinical manifestations of APS. Additional studies to conclusively define the relevance and prognosis impact of testing for these antibodies in the daily routine clinical practice are still required.

When assessing risk, the use of GAPSS may provide valuable information regarding thrombosis or pregnancy loss risk, switching from the concept of aPL as simply diagnostic antibodies to aPL as relevant risk factors for clinical events.
